# Timing of (supplemental) parenteral nutrition in critically ill patients: a systematic review

**DOI:** 10.1186/s13613-014-0031-y

**Published:** 2014-10-02

**Authors:** Rianne BC Bost, Dave HT Tjan, Arthur RH van Zanten

**Affiliations:** 1 Department of Intensive Care Medicine, Gelderse Vallei Hospital, Willy Brandtlaan 10, Ede, 6716 RP, The Netherlands; 2 Medical Manager Care Division, Intensive & Medium Care, Gelderse Vallei Hospital, Willy Brandtlaan 10, Ede, 6716 RP, The Netherlands

**Keywords:** Critically ill patient, ICU, Parenteral nutrition, Supplemental parenteral nutrition, Timing, Mortality, Nutritional support, Mechanical ventilation, Renal replacement therapy, Muscle wasting

## Abstract

Supplemental parenteral nutrition (SPN) is used in a step-up approach when full enteral support is contraindicated or fails to reach caloric targets. Recent nutrition guidelines present divergent advices regarding timing of SPN in critically ill patients ranging from early SPN (<48 h after admission; EPN) to postponing initiation of SPN until day 8 after Intensive Care Unit (ICU) admission (LPN). This systematic review summarizes results of prospective studies among adult ICU patients addressing the best timing of (supplemental) parenteral nutrition (S)PN. A structured PubMed search was conducted to identify eligible articles. Articles were screened and selected using predetermined criteria and appraised for relevance and validity. After critical appraisal, four randomized controlled trials (RCTs) and two prospective observational studies remained. One RCT found a higher percentage of alive discharge from the ICU at day 8 in the LPN group compared to EPN group (*p* = 0.007) but no differences in ICU and in-hospital mortality. None of the other RCTs found differences in ICU or in-hospital mortality rates. Contradicting or divergent results on other secondary outcomes were found for ICU length of stay, hospital length of stay, infection rates, nutrition targets, duration of mechanical ventilation, glucose control, duration of renal replacement therapy, muscle wasting and fat loss. Although the heterogeneity in quality and design of relevant studies precludes firm conclusions, it is reasonable to assume that in adult critically ill patients, there are no clinically relevant benefits of EPN compared with LPN with respect to morbidity or mortality end points, when full enteral support is contraindicated or fails to reach caloric targets. However, considering that infectious morbidity and resolution of organ failure may be negatively affected through mechanisms not yet clearly understood and acquisition costs of parenteral nutrition are higher, the early administration of parenteral nutrition cannot be recommended.

## 1 Review

### 1.1 Introduction

Nutritional support in the intensive care unit (ICU) is highly debated as critically ill patients are frequently hypermetabolic, catabolic and at risk of both underfeeding and overfeeding. Enteral nutrition (EN) is frequently recommended over parenteral nutrition (PN) as it may preserve gut mucosal barrier function [1],[2] and has been shown to demonstrate beneficial effects on (gut) immunity. The current literature shows evidence in favour of early enteral nutrition (EEN) commenced within 24 to 48 h after ICU admission [3]. EEN is associated with decreased morbidity (lower infection rates, better wound healing, decreased mechanical ventilation duration, ICU and hospital length of stay and duration of recovery) [4] and even reduced mortality [5]. Therefore, EN is the preferred route over parenteral nutrition whenever EN is possible.

Achieving caloric targets with EN may be challenging in the critically ill. A caloric deficit frequently occurs due to slow intake progression, unnecessary stoppages, delayed gastric emptying, enteral feed intolerance and delays in post-pyloric feeding tube placement [6]. The cumulative deficit or caloric debt has been reported to be associated with adverse clinical outcomes. Villet and co-workers showed that delayed initiation of feeding resulted in a marked cumulative energy debt during the first week after ICU admission associated with an increase in infectious complications, days of mechanical ventilation and length of ICU stay. However, possibly not only energy deficit but also deficient protein intake may be relevant and is suggested to play a role in outcome [7],[8].

In recent guidelines, controversy regarding the timing of supplemental PN (SPN) in ICU patients was found [7]-[9]. The European Society for Clinical Nutrition and Metabolism (ESPEN) guidelines recommend the addition of SPN within 24 to 48 h in patients who are expected to be intolerant to EN within 72 h of admission [7], whereas the American Society for Parenteral and Enteral Nutrition (ASPEN) recommends postponing the initiation of PN until day 8 after ICU admission [9].

SPN is used in a step-up approach when full enteral support is not possible or fails to reach caloric targets. This review based on a systematic literature review summarizes results of trials reported in ICU patients addressing the optimal timing of (S)PN.

### 1.2 Methods

#### 1.2.1 Search strategy

A search was conducted on 4 November 2013 in PubMed to identify all published studies reporting on trials addressing the timing of (supplemental) parenteral nutrition in critically ill adult patients, combining synonyms for domain and determinants: (‘critically ill’ OR ‘Intensive Care’ OR ‘ICU’) AND ((parenteral OR intravenous OR i.v.) AND (feeding OR feedings OR nutrition OR pharmaconutrition)). No limits were used.

Duplicates were excluded and remaining references were screened using titles, abstracts and subsequently full texts. Inclusion criteria were compatible domains and determinants and a full text available in Dutch, English or German languages. Exclusion criteria were animal studies, nonadult studies, systematic reviews, meta-analyses, opinion papers, case reports, and evaluation studies of guidelines. References were reviewed for additional studies.

#### 1.2.2 Critical appraisal

Using standardized criteria [10], all selected articles were appraised on relevance and validity (Table 1). Limitations in studies were evaluated, selected from letters to editors and reported per study.

**Table 1:** Critical appraisal of selected studies

**Study**	**Critical appraisal items**										
	**Relevance**		**Validity**								
	**Domain**	**Determinant**	**Randomization**		**Blinding**	**Standardization**	**Missing data**		**Follow-up**		
			**Allocation**	**Similarity**			**%**	**Reason**	**%**	**Reason**	**ITT analysis**
Bauer 2000 [11]	+	+	+	+	+	+	-	-	−	+	+
Cahill 2011 [12]	+	+	x	x	−	−	-	-	-	-	−
Casaer 2011 [13]	+	+	+	+	+/−	+	-	-	-	-	+
Doig 2013 [14]	+	+	+	+	+/−	+	+	+	+	−	+
Heidegger 2013 [15]	+	+	+	+	+/−	+	-	-	+/−	+/−	+
Kutsogiannis 2011 [16]	+	+	x	x	−	−	-	-	-	-	−

Specification per item: Domain: critically ill adult patients on the ICU? + = yes, − = no. Determinant: parenteral nutrition? + = with PN, − = no PN. Randomization: concealed treatment allocation? + = yes, − = no; similarity subgroups in baseline characteristics? + = yes, − = no. Blinding: + = yes, +/− = partial, − = no. Standardization: + = yes, − = no. Missing data: percentage? + = <5%, +/− = 10%, − = >10%; reason? + = reason given, − = reason not given. Follow-up: percentage? + = <5%, +/− = 10%, − = >10%; reason? + = reason given, +/− = not all reasons given, − = reason not given; ITT analysis? + = yes, − = no. ITT analysis, intention to treat analysis; x, not applicable.

Results are presented as primary end points per study and grouped by relevant end points. No recalculations of statistics of original results were performed.

### 1.3 Results

**Figure 1:**
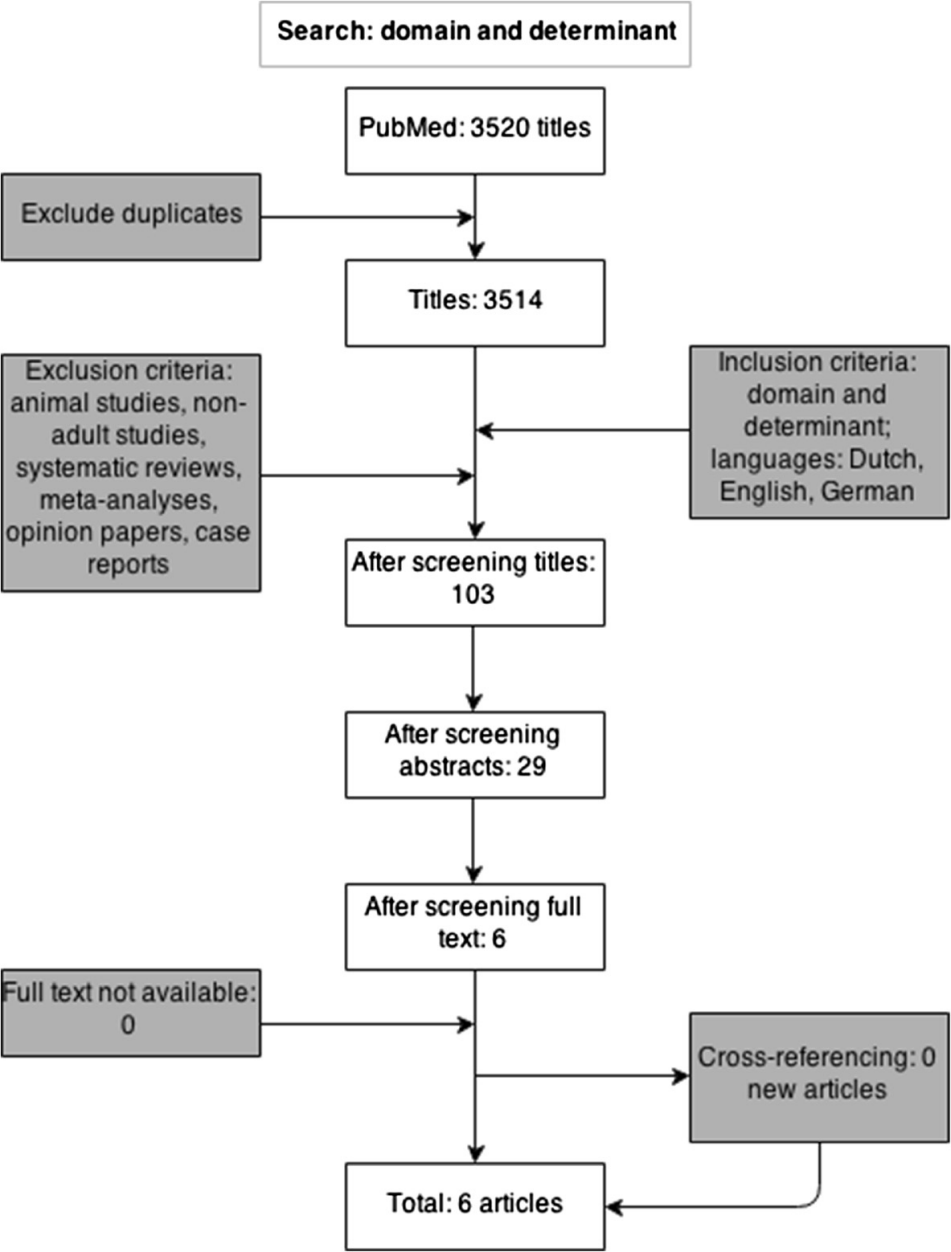
Flow chart of search strategy and selection of articles.

The search strategy yielded a total of 3,520 articles (Figure 1). After the removal of duplicates and screening on predefined criteria, six articles remained (Table 2). Four studies were randomized controlled trials (RCTs) [11],[13]-[15], and two articles were prospective observational studies [12],[16].

**Table 2:** Study characteristics

**Study**	**Design**	***N***	**Medical versus surgical patients**	**Determinants**		
				**Treatment group (*n*)**	**Control group (*n*)**	**Third group (*n*)**
Bauer 2000 [11]	RCT	120	Mixed (exclusion of elective surgery)	EN + EPN for 4 to 7 days (60)	EN + placebo for 4 to 7 days (60)	x
Cahill 2011 [12]	Prospective observational study	703	Medical	EN + EPN <48 h of admission (83)	EN >48 h of admission + no PN (541)	EN + LPN both >48 h of admission (79)
Casaer 2011 [13]	RCT	4,640	Almost 90% surgical patients (58.5% admitted electively)	LPN ≥8 days of admission (2,328)	EPN <48 h of admission (2,312)	x
Doig 2013 [14]	RCT	1,372	Mixed	EPN <24 h of admission (686)	Pragmatic standard care: unfed for 2 to 5 days (686)	x
Heidegger 2013 [15]	RCT	305	Mixed	EN + LPN >3 days of admission (153)	EN at day 1 (152)	x
Kutsogiannis 2011 [16]	Prospective observational study	2,920	Mixed	EN <48 h of admission (2,562)	EN + EPN both <48 h of admission (188)	EN <48 h of admission + LPN >48 h of admission (170)

N, number of patients; RCT, randomized control trial; EN, early enteral nutrition; EPN, early parenteral nutrition; x, not applicable; h, hours; LPN, late parenteral nutrition. NB: studies used different definitions for EPN, LPN and EN.

Baseline characteristics and feeding details of patients included in the studies are shown in Table 3. In the study by Kutsogiannis significant differences between study groups in baseline age (mean age in the EN group 58.4 versus 62.3 years in the EPN group and 56.4 years in the LPN group) and body mass index (BMI) (mean BMI in the EN group 27.2 versus 24.5 kg/m^2^ in the EPN group and 27.0 kg/m^2^ in the LPN group) were observed [16]. In two studies [12],[16], significant differences in baseline admission diagnosis and number of days in hospital before ICU admission were found.

**Table 3:** Baseline characteristics

**Baseline characteristics**	**Study**	**Units**	**Study arms**			**Significance of results**
			**EPN**	**LPN**	**EN**	
Age	Bauer 2000 [11]	Years; mean ± SD	53 ± 18	x	55 ± 18	*p* = ns
	Cahill 2011 [12]		64 ± 15.7	62.8 ± 16.3	59.3 ± 16.8	*p* = 0.095
	Casaer 2011 [13]		64 ± 14	64 ± 15	x	*p* = 0.53
	Doig 2013 [14]		68.4 ± 15.1	x	68.6 ± 14.3	NR
	Heidegger 2013 [15]		x	61 ± 16	60 ± 16	NR
	Kutsogiannis 2011 [16]		62.3 ± 17.9	56.4 ± 17.5	58.4 ± 17.9	*p* = 0.02^
Gender	Bauer 2000 [11]	Male sex; no (%)	40 (66.7)	x	42 (70)	*p* = ns
	Cahill 2011 [12]		54 (65.1)	54 (68.4)	303 (56)	*p* = 0.13
	Casaer 2011 [13]		1,486 (64.3)	1,486 (63.8)	x	*p* = 0.75
	Doig 2013 [14]		400 (58.7)	x	420 (61.6)	NR
	Heidegger 2013 [15]		x	110 (72)	105 (69%)	NR
	Kutsogiannis 2011 [16]		121 (64.4)	105 (61.8)	1,563 (61.0)	*p* = 0.66
BMI	Bauer 2000 [11]	kg/m^2^; mean ± SD	26 ± 5	x	26 ± 5	*p* = ns
	Cahill 2011 [12]	kg/m^2^; mean ± SD	26.1 ± 5.3	26.8 ± 5.6	27.4 ± 7.3	*p* = 0.28
		<25 kg/m^2^; no (%)	41 (49.4)	32 (40.5)	225 (41.6)	*p* = 0.054*
		25 to 30 kg/m^2^; no (%)	26 (31.3)	25 (31.7)	166 (31.3)	
		>30 kg/m^2^; no (%)	16 (19.3)	22 (27.8)	140 (25.9)	
	Casaer 2011 [13]	<25 kg/m^2^; no (%)	988 (42.7%)	1,031 (44.2%)	x	*p* = 0.34
		25 to 30 kg/m^2^; no (%)	852 (36.9)	864 (37.1)	x	
		>30 kg/m^2^; no (%)	472 (20.4)	433 (18.6)	x	
	Doig 2013 [14]	kg/m^2^; mean ± SD	27.9 ± 6.8	x	28.5 ± 6.9	NR
		>30 kg/m^2^; no (%)	190 (27.9)	x	224 (32.8)	
		<18.5 kg/m^2^; no (%)	26 (3.8)	x	20 (2.9)	
	Heidegger 2013 [15]	kg/m^2^; mean ± SD	x	25.4 ± 3.9	26.4 ± 4.6	NR
	Kutsogiannis 2011 [16]	kg/m^2^; mean ± SD	24.5 ± 4.7	27.0 ± 6.9	27. 2 ± 7.0	*p* < 0.0001^
		<25 kg/m^2^; no (%)	120 (63.8)	74 (43.5)	1,143 (44.6)	*p* < 0.0003^
		25 to 30 kg/m^2^; no (%)	47 (25.0)	53 (31.6)	756 (29.7)	
		>30 kg/m^2^; no (%)	21 (11.1)	41 (24.1)	649 (25.3)	
Severity of illness	Bauer 2000 [11]	SAPS II score; mean ± SD	43 ± 14	x	41 ± 13	*p* = ns
	Cahill 2011 [12]	APACHE II score; mean ± SD	25.2 ± 8.5	24.9 ± 8.4	24.3 ± 8.5	*p* = 0.902
	Casaer 2011 [13]	APACHE II score; mean ± SD	23 ± 11	23 ± 10	x	*p* = 0.85
	Doig 2013 [14]	APACHE II score; mean ± SD	20.5 (7.4)	x	21.5 (7.8)	NR
	Heidegger 2013 [15]	SAPS II score; mean ± SD	x	49 ± 17	47 ± 15	NR
		APACHE II score; mean ± SD	x	22 ± 7	23 ± 7	NR
	Kutsogiannis 2011 [16]	APACHE II score; mean ± SD	22.6 ± 8.4	23.3 ± 7.9	22.0 ± 7.9	*p* = 0.11
Amounts of calories delivered	Bauer 2000 [11]	Enteral intake; mean kcal/kg/day on day 7	14.8 ± 4.6	x	13.2 ± 4.3	*p* = 0.6
		Parenteral intake; mean kcal/kg/day on day 7	9.9 ± 3.1	x	1.1 ± 0.3	*p* < 0.0001
		Total intake; mean kcal/kg/day on day 7	24.6 ± 4.9	x	14.2 ± 6.5	*p* < 0.0001
	Cahill 2011 [12]	Enteral intake; mean kcal/kg/day ± SD	5.1 ± 4.9	4.4 ± 3.9	8.8 ± 5.3	*p* < 0.001^†^
		Parenteral intake; mean kcal/kg/day ± SD	12.0 ± 6.3	8.5 ± 5.9	0	*p* < 0.001
		Total intake; mean kcal/kg/day ± SD	17.5 ± 5.8	13.6 ± 6.3	9.9 ± 5.5	*p* < 0.001
	Casaer 2011 [13]	Enteral intake; median kcal/kg/day on day 7, resp day 8	4 vs 4	6 vs 6	x	NR‡
		Parenteral intake; median kcal/kg/day on day 7, resp day 8	20 vs 20	3 vs 4	x	
		Total intake; median kcal/kg/day on day 7	27 vs 26	13 vs 21	x	
	Doig 2013 [14]	Mean kcal/pt/day on day 1, resp day 7	400 vs 1,300	x	0 vs 1,300	NR^‡^
	Heidegger 2013 [15]	kcal/kg/day; mean energy delivery between days 4 and 8 ± SD	x	28 ± 5	20 ± 7	*p* < 0.0001
	Kutsogiannis 2011 [16]	Enteral intake; mean kcal/kg/day ± SD	10.2 ± 6.4	7.3 ± 5.1	14.3 ± 6.5	*p* < 0.001
		Parenteral intake; mean kcal/kg/day ± SD	9.0 ± 5.9	7.6 ± 5.9	0	*p* = 0.02
		Total intake; mean kcal/kg/day ± SD	19.5 ± 6.2	15.7 ± 6.1	15.2 ± 6.5	*p* < 0.001
Amounts of proteins delivered	Bauer 2000 [11]		NR	x	NR	
	Cahill 2011 [12]	Adequacy of protein (%); mean ± SD	71.5 ± 24.9	53.2 ± 22.7	38.7 ± 21.6	*p* < 0.001
	Casaer 2011 [13]		NR	NR	x	
	Doig 2013 [14]		NR	x	NR	
	Heidegger 2013 [15]	g/kg/day; mean protein delivery between day 4 and 8 ± SD	x	1.2 ± 0.2	0.8 ± 0.3	*p* < 0.0001
	Kutsogiannis 2011 [16]	Enteral intake; mean g/kg/day ± SD	0.53 ± 0.33	0.47 ± 0.29	0.77 ± 0.35	*p* < 0.0001
		Parenteral intake; mean g/kg/day ± SD	0.63 ± 0.39	0.71 ± 0.40	0	*p* = 0.48
		Total intake; mean g/kg/day ± SD	0.94 ± 0.40	0.77 ± 0.30	0.77 ± 0.35	*p* < 0.0001
Type of nutrition	Bauer 2000 [11]	EN: modular polymeric diet: protein (20%), polyunsaturated fats (30%), carbohydrates (50%), nonsoluble fibers, sodium chloride (2 g/l), potassium chloride (3 g/l) and a standard solution of hydro- and liposoluble vitamins				
		PN: 3-in-1 solution of carbohydrates, fat and protein, Vitrimix KV and hydrosoluble vitamins, Soluvit				
		Placebo: sodium chloride 0.9% with intralipid 20% (50 ml/l) and Soluvit (10 ml/l)				
	Cahill 2011 [12]	NR				
	Casaer 2011 [13]	EN: mainly Osmolite				
		EPN: intravenous 20% glucose solution on days 1 and 2. On day 3: PN was initiated				
		LPN: intravenous 5% glucose solution. On day 8: PN was initiated				
		PN: trace elements, minerals (potassium, phosphate, magnesium) and vitamins				
	Doig 2013 [14]	Standard care: was defined pragmatically and not via protocol. The attending clinician selected the route, starting rate, metabolic targets and composition of nutrition.				
		PN: standard parenteral nutrition from a ready-to-mix three-chamber bag containing amino acids, glucose, lipids, and electrolytes (Kabiven G19%). Trace elements, minerals and vitamins were added as clinically appropriate.				
	Heidegger 2013 [15]	EN: polymeric, fibre-enriched formulas, containing 1.05 to 1.62 kcal/ml of energy (18% proteins, 29% lipids (8% medium-chain triglycerides), 53% carbohydrates)				
		PN: 0.62 to 1.37 kcal/ml of energy (20% proteins, 29% lipids (15% medium-chain triglycerides), 51% carbohydrates).				
	Kutsogiannis 2011 [16]	NR				

EPN, early parenteral nutrition; LPN, late parenteral nutrition; EN, enteral nutrition; SD, standard deviation; x, not applicable; *p*, *p*-value; ns, nonsignificant; NR, not reported; no, number of patients; %, percentage; kg/m^2^, weight in kilograms divided by height in meters squared; SAPS II score, simplified acute physiology score; APACHE II score, score on the Acute Physiology and Chronic Health Evaluation II (range from 0 to 71, with higher scores indicating a greater severity of illness); kcal, kilocalories; kg, kilograms; resp, respectively; vs, versus; g, grams; PN, parenteral nutrition; l, liters.

NB: studies use different definitions for EPN, LPN and EN. ^, comparing EPN versus EN and EPN versus LPN; *, in this EN group information of 10 patients is missing; ^†^, comparing EPN versus EN and LPN versus EN; ^‡^, these are estimated values from a figure, as no values are given.

Table 4 summarizes the primary end points of the selected studies. Furthermore, in Tables 5, 6, 7, 8 and 9, the categorized end points are shown for mortality, ICU and hospital length of stay, infections, nutrition targets, mechanical ventilation, glucose control, renal replacement therapy, muscle wasting and fat loss.

**Table 4:** Primary end points in included studies and results for EPN, LPN and EN groups

**Primary end points**	**Studies**					
	**RCT**				**Prospective observational study**	
	**Bauer 2000[**[11]**]**	**Casaer 2011[**[13]**]**	**Doig 2013[**[14]**]**	**Heidegger 2013[**[15]**]**	**Cahill 2011 [**[12]**]**	**Kutsogiannis 2011[**[16]**]**
ICU length of stay (median days)	x	3 days (LPN) vs 4 days (EPN); HR 1.06; 95% CI 1.00 to 1.13; *p* = 0.04	x	x	x	x
Mortality; day 60	x	x	22.8% (standard care) vs 21.5% (EPN), RD −1.26%; 95% CI −6.6 to 4.1; *p* = 0.60	x	x	x
Nosocomial infection between days 9 to 28	x	x	x	27% (EN + LPN) vs 38% (EN); HR 0.65, 95% CI 0.43 to 0.97; *p* = 0.0338	x	x
Alive discharge from hospital	x	x	x	x	x	EN + EPN vs EN: HR 0.75; 95% CI 0.59 to 0.96; EN + LPN vs EN: HR 0.64, 95% CI 0.51 to 0.81
Retinol-binding protein (RBP)	Significant increase in EN + EPN vs EN + placebo from day 0 to day 7; *p* = 0.0496	x	x	x	x	x
Prealbumin	Significant increase in EPN vs placebo (day 0 to day 7); *p* = 0.0369	x	x	x	x	x

RCT, randomized control trial; ICU, intensive care unit; x, not investigated as a primary end point; LPN, late parenteral nutrition; EPN, early parenteral nutrition; HR, hazard ratio; 95% CI, 95% confidence interval; *p*, *p*-value; vs, versus; RD, risk difference; EN, enteral nutrition. NB: studies used different definitions for EPN, LPN and EN. In the study by Cahill et al. [12], no primary end point was described.

**Table 5:** Mortality end points in included studies and results for EPN, LPN and EN groups

**Outcome**	**Study**	**Study arms**			**Significance of results**
		**EPN**	**LPN**	**EN**	
Mortality in ICU (%)	Casaer 2011[13]	6.3	6.1	x	*p* = 0.76
	Heidegger 2013 [15]	x	0.5	7.0	*p* = 0.2118
Mortality in hospital (%)	Casaer 2011[13]	10.9	10.4	x	*p* = 0.63
Mortality at discharge (%)	Casaer 2011[13]	28.3	24.8	x	*p* = 0.007 (discharge from ICU)
	Kutsogiannis 2011 [16]	NR	NR	NR	EPN vs EN: HR 0.75; 95% CI 0.59 to 0.96; LPN vs EN: HR 0.64, 95% CI 0.51 to 0.81 (discharge from hospital)
Mortality at day 28 (%)	Heidegger 2013 [15]	x	13.0	18.0	*p* = 0.1193
Mortality at day 60 (%)	Cahill 2011 [12]	42.2	27.9	34.2	*p* = 0.17
	Kutsogiannis 2011 [16]	34.6	35.3	27.8	*p* = 0.02^†^
Mortality at day 90 (%)	Bauer 2000 [11]	28.0	x	30.0	no *p*-value, ns
	Casaer 2011[13]	11.2	11.2	x	*p* = 1.00
Mortality at 2 years (%)	Bauer 2000 [11]	40.0	x	40.0	no *p*-value, ns

EPN, early parenteral nutrition; LPN, late parenteral nutrition; EN, enteral nutrition; ICU, intensive care unit; x, not applicable; *p*, *p*-value; ns, nonsignificant; NR, not reported; vs, versus; HR, hazard ratio; 95% CI, 95% confidence interval. NB: studies used different definitions for EPN, LPN and EN. ^†^, comparing EN versus LPN.

**Table 6:** Length of stay end points in included studies and results for EPN, LPN and EN groups

**Outcome**	**Study**		**Study arms**			**Significance of results**
			**EPN**	**LPN**	**EN**	
ICU length of stay	Bauer 2000 [11]	Mean days	16.9 ± 11.8	x	17.3 ± 12.8	no *p*-value, ns
	Doig 2013 [14]	Mean days	8.6	x	9.3	RD −0.75 (−1.47 to 0.04); *p* = 0.06
	Heidegger 2013 [15]	Mean days	x	13	13	*p* = 0.2592
	Cahill 2011 [12]	Median days	15	22.1	12.4	*p* < 0.001^†^
	Kutsogiannis 2011 [16]	Median days	13.9	18.3	11.7	*p* = 0.003^
Hospital length of stay	Bauer 2000 [11]	Mean days	31.2 ± 18.5	x	33.7 ± 27.7	*p* = 0.0022
	Casaer 2011 [13]	Median days	16	14	x	HR 1.06; 95% CI 1.00 to 1.13; *p* = 0.04.
	Doig 2013 [14]	Mean days	25.4	x	24.7	RD 0.7; 95% CI −1.4 to 3.1; *p* = 0.50
	Heidegger 2013 [15]	Mean days	x	31	32	*p* = 0.8781
	Cahill 2011 [12]	Median days	47.6	35	24.8	*p* < 0.001
	Kutsogiannis 2011 [16]	Median days	33.4	35.3	27.1	*p* = 0.004

EPN, early parenteral nutrition; LPN, late parenteral nutrition; EN, enteral nutrition; ICU, intensive care unit; x, not applicable; *p*, *p*-value; ns, nonsignificant; RD, risk difference; HR, hazard ratio; 95% CI, 95% confidence interval. NB: studies used different definitions for EPN, LPN and EN. ^†^, comparing EPN versus EN and comparing LPN versus EN. ^, comparing EN versus LPN.

**Table 7:** Infection end points in included studies and results for EPN, LPN and EN groups

**Outcome**	**Study**	**Study arms**			**Significance of results**
		**EPN**	**LPN**	**EN**	
Infections (%)	Bauer 2000 [11]	47.0	x	38.0	Respiratory infections: no *p*-value, ns
	Bauer 2000 [11]	18.0	x	27.0	UTI: no *p*-value, ns
	Casaer 2011 [13]	26.2	22.8	x	*p* = 0.008
	Doig 2013 [14]	NR	x	NR	No significant differences between standard care or EPN
Antibiotic (mean days ± SD)	Heidegger 2013 [15]	x	5 ± 7	6 ± 7	*p* = 0.0298
Antibiotic free (mean days ± SD)	Heidegger 2013 [15]	x	15 ± 9	13 ± 10	*p* = 0.0126^†^

EPN, early parenteral nutrition; LPN, late parenteral nutrition; EN, enteral nutrition; *p*, *p*-value; ns, nonsignificant; UTI, urinary tract infections; NR, not reported; SD, standard deviation. NB: studies used different definitions for EPN, LPN and EN. ^†^, analyses performed between day 9 and day 28.

**Table 8:** Nutritional adequacy in included studies and results for EPN, LPN and EN groups

**Outcome**	**Study**	**Study arms**			**Significance of results**
		**EPN**	**LPN**	**EN**	
Adequacy of calories (mean% ± SD)	Cahill 2011 [12]	74.1 ± 21.2	57.4 ± 22.7	42.9 ± 21.2	*p* < 0.001^†^
	Kutsogiannis 2011 [16]	81.2 ± 23.1	64.3 ± 20.6	63.4 ± 23.4	*p* < 0.0001^†^
Adequacy of proteins (mean% ± SD)	Cahill 2011 [12]	71.5 ± 24.9	53.2 ± 22.7	37.8 ± 21.6	*p* < 0.001^†^
	Kutsogiannis 2011 [16]	80.1 ± 30.3	59.9 ± 21.2	59.3 24.3	*p* < 0.0001^†^
Mean energy delivery (kcal/kg/d ± SD; % of energy target ± SD)	Heidegger 2013 [15]	x	28 ± 5; 103 ± 18	20 ± 7; 77 ± 27	*p* < 0.0001^

EPN, early parenteral nutrition; LPN, late parenteral nutrition; EN, enteral nutrition; %, percentage, SD, standard deviation; *p*, *p*-value; kcal, kilocalorie; kg, kilogram; d, day; x, not applicable. NB: studies used different definitions for EPN, LPN and EN. ^†^, comparing EPN versus EN and comparing LPN versus EN and comparing EPN versus LPN; ^, analyses between day 4 and day 8.

**Table 9:** Remaining secondary end points in included studies and results for EPN, LPN and EN groups

**Outcome**	**Study**		**Study arms**			**Significance of results**
			**EPN**	**LPN**	**EN**	
Mechanical ventilation	Bauer 2000 [11]	Mean days	11 ± 9	x	10 ± 8	no *p*-value, ns
	Casaer 2011 [13]	Median days	2	2	x	*p* = 0.02
		Duration >2 days	40.2%	36.3%	x	*p* = 0.006; in LPN 9.7% relative reduction in mechanical ventilation in patients requiring ≥2 days mechanical ventilation
	Doig 2013 [14]		NR	x	NR	EPN: 1.07 fewer days than standard care.
		Mean days per 10 patients x ICU days	7.73	x	7.26	MD −0.47; 95% CI −0.82 to −0.11; *p* = 0.01)
	Heidegger 2013 [15]	Mean hours (95% CI)	x	153 (126 to 178)	166 (138 to 189)	*p* = 0,2912
	Cahill 2011 [12]	Median days	8.8	18.2	9.3	*p* < 0.001^†^
	Kutsogiannis 2011 [16]	Median days	9.1	14.5	8.4	*p* = 0.007^
Hypoglycaemia	Bauer 2000 [11]	Mean glucose in grams per liter at day 7 (±SD)	1.16 ± 0.56	x	1.31 ± 0.62	*p* < 0.05
	Casaer 2011 [13]	Number of patients (%)	45 (1.9)	81 (3.5)	x	*p* = 0.001
	Heidegger 2013 [15]	NR	x	NR	NR	No difference between EN + LPN vs EN
Duration of RRT	Bauer 2000 [11]	Mean days (±SD)	0.8 (±2.4)	x	0.9 (±2.3)	No *p*-value, ns
	Casaer 2011 [13]	Median days	10	7	x	*p* =0.008
	Heidegger 2013 [15]		x	NR	NR	No difference between EN + LPN vs EN
Body composition (score increase/week)	Doig 2013 [14]	Increased SGA score/week	0.43	x	0.27	MD −0.16; 95% CI −0.28 to −0.038; *p* = 0.01
		Increased SGA score/week	0.44	x	0.31	MD −0.13; 95% CI −0.25 to −0.01; *p* = 0.04

EPN, early parenteral nutrition; LPN, late parenteral nutrition; EN, enteral nutrition; x, not applicable; *p*, *p*-value; ns, nonsignificant; NR, not reported; ICU, intensive care unit; MD, mean difference; 95% CI, 95% confidence interval; vs, versus; RRT, renal replacement therapy; SD, standard deviation; SGA, Subjective Global Assessment. NB: studies used different definitions for EPN, LPN and EN. ^†^, comparing EPN versus EN and comparing LPN versus EN. ^, comparing EN versus LPN.

#### 1.3.1 Mortality

Results are shown in Tables 4 and 5. Casaer [13] reported a higher percentage of alive discharge from ICU after day 8 in the LPN group compared to EPN (*p* = 0.007) but no difference in ICU and in-hospital mortality. None of the other RCTs [11],[14],[15] observed differences in ICU or in-hospital mortality rates. Even after a follow-up of 2 years no mortality differences were found.

Kutsogiannis [16] found a higher mortality rate in LPN compared to EN, and the rate of patients discharged alive from hospital was lower in the group that received SPN compared to EN. However, in this study, no mortality analysis was performed comparing EPN and LPN.

### 1.4 ICU length of stay

Results are shown in Tables 4 and 6. All studies [11]-[16] examined ICU length of stay and hospital length of stay resulting in contradictory results. In three studies [11],[14],[15], there was no difference in ICU length of stay among all study groups. The other three studies reported results that are more difficult to interpret. Casaer reported an increased length of stay in the ICU in the EPN group compared to LPN (median 4 versus 3 days, respectively) [13]. Cahill and Kutsogiannis found a shorter stay in the EN group, compared to the PN groups. However, no analysis was performed comparing EPN and LPN [12],[16].

### 1.5 Hospital length of stay

Results are shown in Tables 4 and 6. Hospital length of stay was significantly prolonged in the EPN group compared to the LPN group in the study by Casaer [13]. However, Bauer [11] found a significantly shorter hospital length of stay in the EPN group. Both Cahill [12] and Kutsogiannis [12],[16] found a shorter length of stay in the hospital in patients not receiving any form of PN. They did not compare EPN with LPN. In the other studies, there was no difference in hospital length of stay [14],[15].

#### 1.5.1 Infections

Results are shown in Tables 4 and 7. Rates of nosocomial infections were higher in patients receiving EPN in the study by Casaer compared to LPN (*p* = 0.008) [13]. Heidegger showed significantly lower infection rates in the PN group (27% [41/153]) compared with EN (38% [58/152]) (hazard ratio (HR) 0.65 (95% CI 0.43 to 0.97); *p* = 0.0338) [15]. In addition, the number of antibiotic days was found significantly less in the PN group. However, Heidegger reported data on new infections after day 9, whereas randomization took place after day 3. SPN was provided on day 4, and the SPN group had more total infectious events between day 4 and day 8 (34% [52/153] versus 28% [43/152]). Total infections between day 4 and day 28 are similar (114/153 versus 100/152) and nonsignificantly different [17]-[19].

No differences were found in infection rates in patients receiving EPN in other studies [11],[14].

#### 1.5.2 Nutrition targets

Results are shown in Table 8. The studies by Cahill and Kutsogiannis found a more adequate intake of calories in the EPN group (74.1% and 81.2%, respectively; *p* < 0.0001) compared to the LPN group (57.4% and 64.3%, respectively; *p* < 0.0001) [12],[16]. Similar results were found when addressing the adequacy of protein intake (71.5% and 80.1%, respectively, in the EPN group compared to 53.2% and 59.9%, respectively, in the LPN group; *p* < 0.0001). Heidegger [15] observed a higher rate of patients reaching their calculated energy target in the PN group compared to the EN group (mean energy delivery as percentage of target was 103% and 77%, respectively; *p* < 0.0001).

#### 1.5.3 Mechanical ventilation

Results are shown in Table 9. Duration of mechanical ventilation data showed divergent results among studies. When EN in combination with EPN is compared to EN with a placebo, no differences were found [11], but comparing EPN with standard care showed a shorter duration of mechanical ventilation (0.47 days) in favour of EPN (*p* = 0.01) [14]. In contrast, in LPN patients, Casaer reported a relative reduction of mechanical ventilation duration (9.7%) among patients requiring ≥2 days of mechanical ventilation compared with EPN [13].

LPN was associated with a longer duration of mechanical ventilation in both observational studies when compared with EN (18.2 versus 9.3 median days; *p* < 0.001 [12] and 14.5 versus 8.4 median days; *p* = 0.007 [16]). However, they did not compare EPN and LPN. Furthermore, the study by Heidegger showed no differences in mechanical ventilation duration comparing LPN to EN (mean 153 versus 166 h; *p* = 0.2912 [15]).

### 1.6 Renal replacement therapy

Results are shown in Table 9. Casaer [13] found a significant shorter duration of renal replacement therapy (RRT) among patients on LPN compared with EPN (median 7 versus 10 days, *p* = 0.008). Other studies investigating RRT duration did not find significant differences [11],[15].

#### 1.6.1 Glucose control

Results are shown in Table 9. In one study glucose control was more strict in the EPN than in the placebo group (*p* = 0.0392) [11]. Hypoglycaemia more often occurred in the LPN patients compared with EPN patients (3.5% versus 1.9%; *p* = 0.001) in one study [13]; however, no differences in hypoglycaemia were found in another [15].

### 1.7 Muscle wasting and fat loss

Results are shown in Table 9. Doig [14] reported less muscle wasting (0.43 versus 0.27 score increase per week; mean difference −0.16; 95% CI −0.28 to −0.038; *p* = 0.01) and fat loss (0.44 versus 0.31 score increase per week; mean difference −0.13; 95% CI −0.25 to −0.01; *p* = 0.04) based on Subjective Global Assessment in the EPN group compared with standard care.

### 1.8 Discussion

We reviewed relevant studies on early or late SPN in critically ill adult patients. Results should be interpreted with caution as study populations differed markedly (medical versus surgical versus mixed) and often different primary and secondary end points were addressed. Furthermore, definitions for early and late SPN varied markedly. For this reason, we decided not to provide forest plots of results.

#### 1.8.1 Mortality

Mortality rates at various end points showed no significant differences between EPN and LPN groups. Follow-up duration in the selected studies varied from 28 days until 2 years. This may have resulted in incorrect lower ICU or hospital mortality rates in studies with shorter follow-ups, as some patients still may have been hospitalized at day 28. However, early SPN does not show to reduce mortality.

#### 1.8.2 Morbidity

Commencing early PN does not result in benefits in ICU or hospital length of stay. Duration of renal replacement therapy may increase by EPN. Effects on duration of mechanical ventilation are contradictory. Doig [14] showed that EPN resulted in significantly fewer days of invasive ventilation; however, the mean reduction of ventilation duration was only 0.47 days and could not be translated into a significantly shorter ICU or hospital length of stay. In contrast, Casaer showed that more patients needed prolonged ventilation in the EPN group [13].

Infections reported after randomization were either nonsignificant or increased during EPN. The lower number of antibiotic days and more antibiotic-free days in the EPN group versus the LPN group in the study by Heidegger may be explained by either a lower total number of infections reported in the EPN group or only reporting infections after day 9 (and randomization occurred at day 3). Early SPN does not reduce infectious morbidity and even seems to increase infection rates.

### 1.9 Nutritional intake, metabolic consequences and effects on body composition

Three studies examined the energy, calorie and protein delivery [12],[15],[16] and found that PN promotes reaching energy and protein targets. Within the PN groups, EPN resulted in more optimal intake of calories and proteins, suggesting better feeding adequacy compared with LPN. Strikingly, these findings do not translate into marked differences in end points.

Effects on glucose regulation and incidence of hypoglycaemia were not consistent. Other studies have demonstrated clearly that parenteral nutrition negatively affects insulin sensitivity and that parenteral-nutrition-induced hyperglycaemia is associated with increased morbidity and mortality [20].

One study reported EPN to be protective against both muscle wasting and fat loss. Although this might be expected to translate into improved recovery of physical function, mortality and length of stay obtained 60 days after enrolment did not differ between groups [14]. Meanwhile, a preplanned substudy of the EPaNIC study showed that early parenteral nutrition did not prevent the wasting of skeletal muscle in critical illness and increased the amount of adipose tissue within the muscle compartments [21].

#### 1.9.1 Economic consequences

In general, acquisition costs for parenteral nutrition are higher compared with enteral nutrition [11],[22].

Doig and co-workers showed a small reduction in duration of mechanical ventilation in patients on EPN [23]. This incurred lower costs estimated at US$3,150 per patient (95% confidence interval US$1,314 to US$4,990) [23]. In contrast, Casaer and co-workers showed significantly higher expenditures up to a mean total cost increase of 1,210.00 EUR/patient (*p* = 0.02) by EPN, when incorporating the full PN costs. This was mainly due to the acquisition costs of PN and anti-infective medications [24].

We believe that a reasonable interpretation of the present evidence is that EPN in adult critically ill patients does not confer clinically relevant benefits compared to LPN with respect to morbidity and mortality end points. Some studies report small benefits, others no effect or even increased morbidity.

Patients that reach energy and protein targets by the enteral route show better outcomes than patients that did not [25],[26], whereas reaching these targets through addition of PN seems to provide different and potentially less beneficial effects. Clearly, providing calories and proteins through EN or PN induces divergent effects.

At present, it is unclear which factors induce the negative pathophysiological effects of early administration of PN that counterbalance the beneficial effect of a more optimal nutritional intake with respect to calories and proteins. Potentially, SPN may interfere with the early inflammatory response present in most ICU patients. Among negative effects, autophagy has been suggested. Autophagy is the basic catabolic mechanism that involves cell degradation of unnecessary or dysfunctional cellular components through the actions of lysosomes. A planned subanalysis of the EPaNIC trial [27] found that tolerating a substantial macronutrient deficit early during a critical illness did not affect muscle wasting but allowed more efficient activation of autophagic quality control of myofibres and reduced weakness. Thus, SPN may hamper the autophagy phenotype. The role of autophagy prevention in critically ill patients has to be determined in the future. Other suggestive negative effects of SPN are increased risk of overfeeding and refeeding, fat overload, glucose intolerance and immune-modulating effects of lipids [28].

#### 1.9.2 Study limitations

Several limitations in the selected studies were found. Two studies [12],[16] were not randomized controlled trials; therefore, inclusion bias may have occurred, treatment was not blinded and we found statistically significant differences in baseline characteristics. Furthermore, results were based on partly overlapping study groups. In the Cahill study [12], all surgical patients were excluded to select a more homogenous study population, as surgical patients typically are more difficult to feed, have lower tolerance to EN, and receive less nutrition compared to medical patients. This reduces external validity for surgical ICU patients. Furthermore, both groups receiving PN (EPN versus LPN) were relatively small potentially causing insufficient power to detect clinically relevant differences.

The study by Heidegger and co-workers [15] was heavily criticized for performing analyses on new infections between days 9 and 28, whereas randomization occurred on day 3. The registered protocol indicated that the primary outcome was the infection rate in the first 28 days, not just between day 9 and day 28. SPN was provided on day 4, and the SPN group had more total infectious events between day 4 and day 8 (34% [52/153] versus 28% [43/152]). Total infections between day 4 and day 28 appear quite similar (114 versus 100). Selective outcome bias seems to be present [17]-[19].

Then, the authors state that 25 to 30 kcal/kg per day was the target for adequate energy intake. But control patients received only 20 kcal/kg per day, potentially increasing the risk of underfeeding-related complications, such as infections. The intervention group, which received 28 kcal/kg per day through enteral nutrition plus 10 kcal/kg per day parenterally, showed a significantly lower rate of infections compared with the control group receiving 20 kcal/kg per day. The authors conclude in favour of the parenteral supplementation; however, observed differences might be only related to a higher calorie intake [29].

The study performed by Doig [14] was prematurely terminated for financial reasons. This has changed the period of follow-up and therefore might have influenced the presented results.

Finally, in the study by Casaer [13], almost 90% of the patients studied were surgical patients, the majority of whom (58.5%) appeared to be admitted electively. Study patients remained in the ICU for a limited time, with more than 70% of subjects averaging only a 3- to 4-day length of stay. These patients were only moderately severely ill, with an 8% ICU mortality. Therefore, this could suggest that external validity for critically ill patients with higher severity of illness and medical patients is limited; however, a *post-hoc* sensitivity analysis addressing the effects of severity of illness did not impact on the reported results [30]. Furthermore, it is hard to attribute all adverse events in this study to early PN, when the majority of study patients received limited exposure to early PN and more than 70% of the late PN group did not receive PN at all. It is therefore conceivable that benefits of late PN were seen because the majority of patients received no PN or early high-glucose loading. Patients randomized to the EPN group received glucose 20% at 40 ml/h on the admission day, in contrast to patients randomized to the LPN group. They received a volume of glucose 5% that was required to obtain adequate hydration taking into account the volume of EN that was being delivered [31]. In addition, all patients in both groups were managed by tight glucose control, using the protocol reported by Van den Berghe [32],[33]. This concept of tight glucose control has subsequently been shown to be ineffective and potentially harmful [34]. It is unclear how this glucose loading and tight glucose regulation strategy impact on the reported effects of early and late SPN.

Other methodological issues may have influenced reported results such as incorrect interpretation of data due to inappropriate censoring for time-to-event analyses when the duration of follow-up is not identical for all subjects. This could be relevant for end points such as ICU and hospital length of stay or hospital mortality. This problem can be circumvented by reporting landmark time-to-event analysis (for example, 180-day mortality) in all patients [35]. As this has not been done in many studies, inappropriate censoring may be present and may invalidate reported data.

Strengths of this systematic review are the large number of patients studied in the selected studies (*n* = 10,060) and the structured analysis and appraisal of the available literature.

However, several limitations of this systematic review also have to be mentioned. Definitions of EPN and LPN are different among studies. When the ‘early’ period stops and the ‘late’ period starts is not defined. Late may be considered at the end of the first week [36]. Furthermore, the study design, year of patient inclusion and primary and secondary end point variations may affect interpretation of overall results. Moreover, insufficient information is available on vitamins, trace elements and parenteral lipids used.

## 2 Conclusions

In adult critically ill patients, when full enteral nutrition support is not possible or fails to reach caloric targets, early administration of supplemental parenteral nutrition compared with late administration (at the end of the first week after ICU admission) does not confer major benefits with respect to morbidity and mortality. However, considering that infectious morbidity and resolution of organ failure may be negatively affected through mechanisms not yet clearly understood and acquisition costs of parenteral nutrition are higher compared with enteral nutrition, the early administration of parenteral nutrition cannot be recommended. Additional research is warranted to recommend the optimal timing of SPN in critically ill adults.

## Abbreviations

ASPEN: American Society for Parenteral and Enteral Nutrition

EEN: Early enteral nutrition

EN: Enteral nutrition

EPN: Early parenteral nutrition

ESPEN: European Society for Clinical Nutrition and Metabolism

ICU: Intensive care unit

LPN: Late parenteral nutrition

PN: Parenteral nutrition

RCT: Randomized controlled trial

RRT: Renal replacement therapy

(S)PN: (supplemental) parenteral nutrition

## Competing interests

RB and DT declare no competing interests. AVZ has received honoraria for advisory board meetings, lectures and travel expenses from Abbott, Baxter, Danone, Fresenius Kabi, Nestlé, Novartis and Nutricia. No funding was utilized in the preparation of this manuscript. There was no administrative, technical or material support.

## Authors’ contributions

RB designed the study concept, acquired data and drafted the manuscript. DT participated in the design of the study and helped draft the manuscript. AVZ participated in the design of the study, the acquisition of data and drafted the manuscript. All authors had full access to all of the data in the study and take responsibility for the integrity of the data and the accuracy of the data analysis. All authors have revised the manuscript critically for important intellectual content and approved the final manuscript.
